# Middle mesenteric artery: Angiographic and three-dimensional computed tomography findings

**DOI:** 10.1016/j.radcr.2022.03.113

**Published:** 2022-05-09

**Authors:** Mika Muraki, Tomohisa Moriya, Yoshiaki Katada, Ryuhei Masuno, Shuji Suzuki, Shinji Sugahara, Kazuhiro Saito

**Affiliations:** a Department of Radiology, Tokyo Medical University Ibaraki Medical Center, 3-20-1, Chuo, Inashiki-gun Ami-machi, Ibaraki 300-0395, Japan; b Department of Gastroenterological Surgery, Tokyo Medical University Ibaraki Medical Center, Ibaraki, Japan; c Department of Radiology, Tokyo Medical University, Tokyo, Japan

**Keywords:** Middle mesenteric artery, Middle colic artery, Angiography, Computed tomography

## Abstract

The middle mesenteric artery, also known as the third mesenteric artery, is a very rare anomaly. Several anatomical variations of middle mesenteric artery have been reported; in these reports, the right colic artery and/or middle colic artery often originate directly from the aorta. Here, we report a middle mesenteric artery in which the middle colic artery originated directly from the abdominal aorta. We also provide three-dimensional computed tomography and angiography findings and discuss anatomical and embryological considerations.

## Introduction

Middle mesenteric artery (MMA) has been defined as a mesenteric artery originating from the abdominal aorta between the superior mesenteric artery (SMA) and the inferior mesenteric artery (IMA). Many variations in the origins of mesenteric arteries have been reported, but an anomalous origin directly from the abdominal aorta is extremely rare. MMA was first described in 1963 as “double inferior mesenteric arteries” [1] and was named in 1987 [2]. As mentioned above, MMA originates directly from the aorta between the SMA and the IMA. In some cases, the MMA mainly feeds the transverse colon; however, many MMA variations exist [1], [2], [3], [4], [5], [6], [7]. Here, we report an MMA that fed the transverse and proximal descending colon, along with 3-dimensional computed tomography (3D-CT) and angiography findings and embryological considerations.

## Case report

**Fig. 1:**
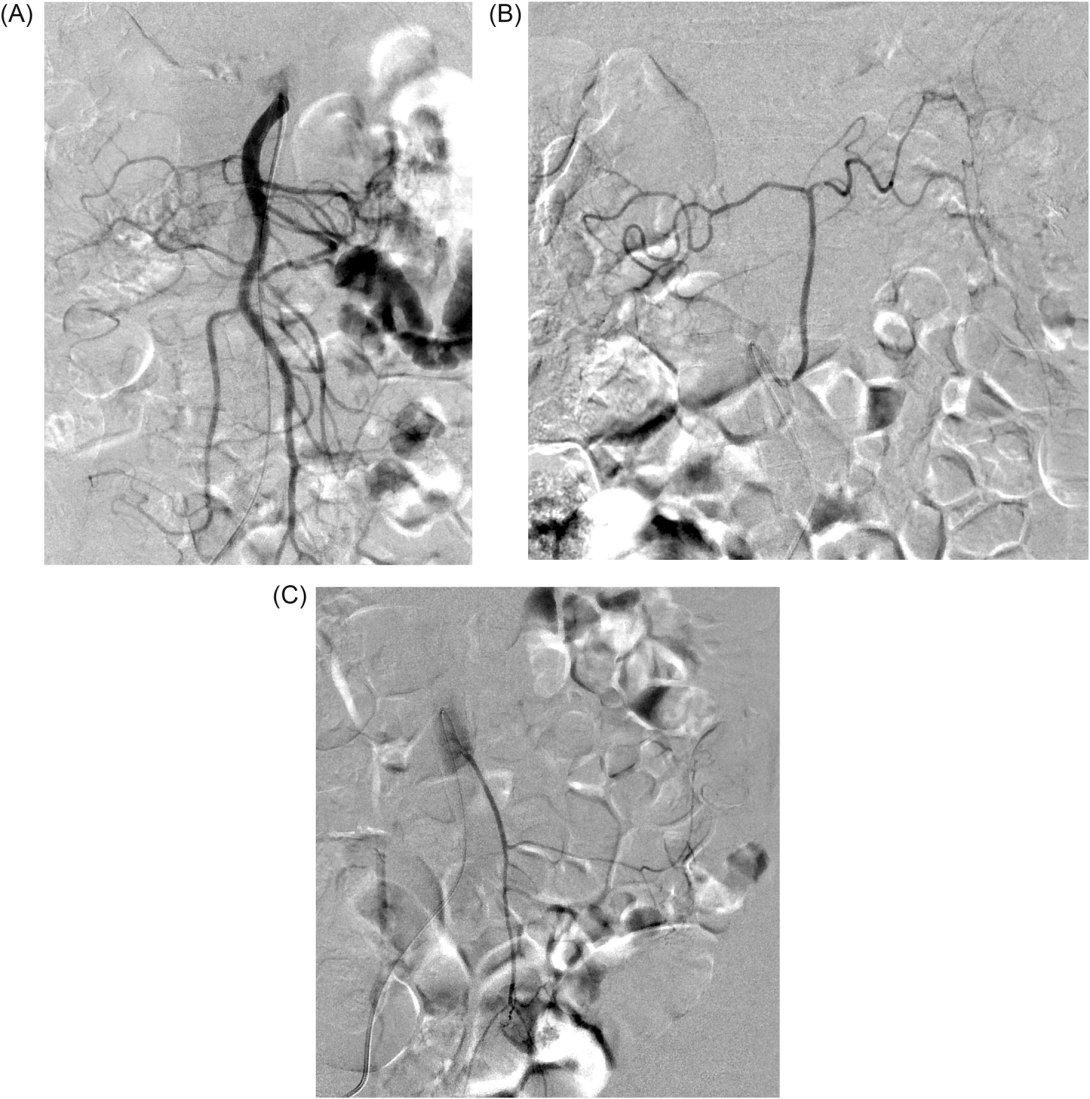
(A) Superior mesenteric artery angiogram. No artery was shown coursing to the transverse colon from any branch of SMA. (B) Selective angiogram of the middle mesenteric artery. The area from the hepatic flexure to the splenic flexure is supplied so-called the middle colic artery. (C) Inferior mesenteric artery originates from the aorta at its normal position. SMA, superior mesenteric artery.

**Fig. 2:**
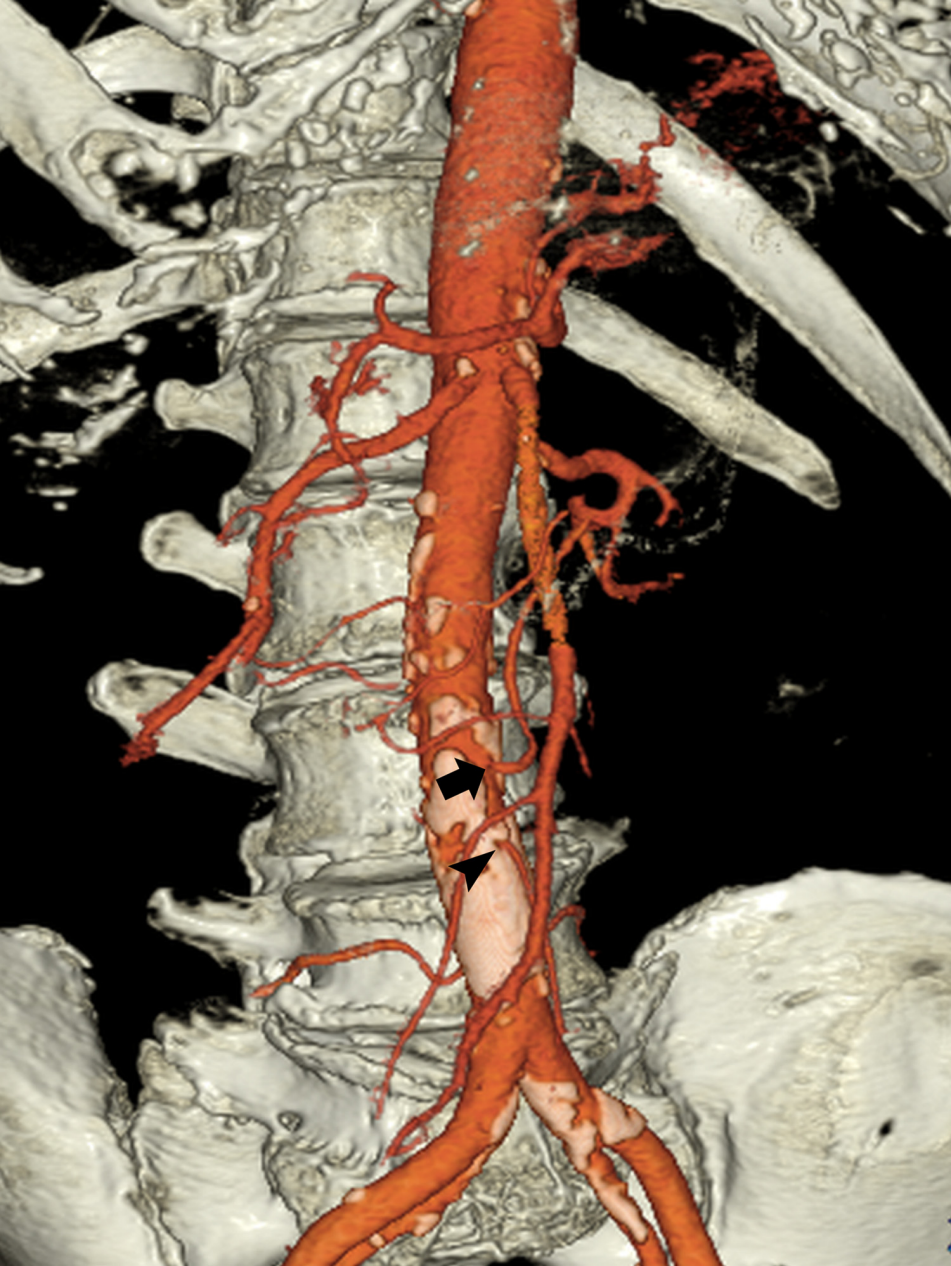
3D-computed tomography. The middle mesenteric artery (arrow) originates from 12 mm cranial to IMA (arrow head). IMA, inferior mesenteric artery.

The case was an 87-year-old man with no history of abdominal surgery or colon disease. He complained of a sudden left abdominal pain and was taken to our hospital as an emergency admission. An abdominal CT showed a hematoma in the retroperitoneal space on the dorsal side of the descending colon. An extravasation in the hematoma and a decrease in the hemoglobin level were observed, and we decided to perform urgent arterial embolization. Anomalous blood vessel originating directly from the aorta that differed from the celiac trunk, SMA and IMA was observed at the time of angiography (Fig. 1A-C). This vessel was located 72 mm caudal to the SMA and 12 mm cranial to the IMA and originated directly from the abdominal aorta at the L3 vertebra level, then ascended the ventral side of the aorta on 3D-CT (Fig. 2). The artery fed the transverse and proximal descending colon similar to the so-called middle colic artery; no middle colic branch was observed in the SMA (Fig. 1A,B).

No extravasation could be detected on the emergent angiography, and the conservative therapy was selected. However, due to colonic ischemia caused by compression of the colon wall by the hematoma, a partial colon resection was performed. The postoperative course was good, and the patient was discharged without any complications.

## Discussion

Embryologically, the development of the mesenteric artery begins at the ventral branch, which is distributed in the yolk sac among the dorsal branch, lateral branch and ventral branch arising from paired dorsal aortas. Then, the paired dorsal aortas fuse, and the ventral branches of paired arteries join to became an unpaired artery. Many anastomoses are formed between these ventral segmental arteries, and many anastomotic vessels disappear as they grow. According to a report by Tandler et al. [4], [5], [6], the 10th ventral segmental artery is formed as a gastric artery, the 11th is formed as a splenic artery, and the 12th is formed as a hepatic artery. The proximal part of the 11th-12th branch disappears, and the celiac trunk is formed from the proximal part of 10th ventral segmental artery, while the SMA is formed from the 13th ventral segmental artery. The inferior mesenteric artery is formed by the fusion of 21st-22nd branches [6]; however, few reports have detailed the development of the IMA, compared with those for the celiac trunk and SMA. Although a relatively large number of anomalies have been reported for the middle colic artery, reports of a so-called MMA originating directly from the aorta are extremely rare.

MMA was first reported by Benton and Cotter in 1963, who described the anomaly as “double inferior mesenteric arteries” [1]. They reported the presence of two inferior mesenteric arteries, one branching from the cranial side and feeding the upper part of the descending colon and one feeding the entire transverse colon, similar to the anomaly where a so-called middle colic artery originates from the aorta. The SMA did not have a vessel corresponding to the middle colic artery, and a marginal artery was found throughout the colon.

In 1987, Lawdahl and Keller were the first to use “MMA” to describe this anomaly [2]. In their case, the MMA supplied the distal transverse colon and the proximal descending colon, similar to cases in which the left colic artery originates directly from the aorta. The middle colic artery has been confirmed to originate from the SMA, which is a different type of anomaly from that reported by Benton and Cotter.

According to a report by Yoshida et al. [7], the MMA branches into two vessels: the right branch feeds the transverse colon, and the left branch feeds the left side of the transverse colon, the splenic flexure, and the proximal descending colon. The middle colic branch of the SMA is absent; and this description is like that reported by Benton and Cotter et al. [1]. According to a report by Koizumi et al. [3], the MMA is divided into four branches: the ileocolic artery, the right colic artery, the middle colic artery, and an accessory middle colic artery. This MMA ran from the cecum and ascending colon to feed the entire transverse colon and splenic flexure. The ileocolic artery, the right colic artery, and the middle colic artery were not observed during SMA angiography, and their report was the first reported case in which the ileocolic artery, the right colic artery, and the middle colic artery formed a common trunk and branched directly from the aorta. In the presently reported case, the middle colic artery was not observed during SMA angiography, and the transverse and upper descending colon were fed by the MMA. Therefore, our case is like the reports by Benton et al. [1] and Yoshida et al. [7], in which the middle colic artery originated directly from aorta. In addition, several anomalies related to the colic branches have also been reported, and various types of anomalies are considered to occur during the fusion and disappearance of the ventral segmental arteries of the 14th to 22nd branches in the SMA and IMA regions, as well as the celiac trunk.

As mentioned in previous reports, the possibility that an MMA might be present must be kept in mind if a colic branch is absent during SMA angiography. Consideration that these anomalous arteries might be present is also important during preoperative 3D-CT examinations of the colonic region.

## Patient consent

Patient consent has been obtained for the publication of this article.
